# Brown adipocyte exosome - derived C22:6 inhibits the IL-1β signaling pathway to alleviate rheumatoid arthritis

**DOI:** 10.3389/fimmu.2025.1543288

**Published:** 2025-05-09

**Authors:** Rui Jiang, Yuanyuan Huang, Rongcai Ye, Yujian Zhang, Meng Dong, Hanlin Zhang, Ziyu Cheng, Zhi Zhang, Jiaqi Zhang, Qiaoli Zhang, Gang Sun, Wanzhu Jin

**Affiliations:** ^1^ State Key Laboratory of Animal Biodiversity Conservation and Integrated Pest Management, Institute of Zoology, Chinese Academy of Sciences, Beijing, China; ^2^ The Zhongzhou Laboratory for Integrative Biology of Henan University, Zhengzhou, Henan, China; ^3^ College of Life Sciences, University of Chinese Academy of Sciences, Beijing, China; ^4^ Department of Gastroenterology and Hepatology, First Medical Center, Chinese People’s Liberation Army General Hospital, Beijing, China; ^5^ Department of Obstetrics and Gynecology, Peking Union Medical College Hospital, Peking Union Medical College, Chinese Academy of Medical Sciences, Beijing, China

**Keywords:** rheumatoid arthritis, exosome, C22:6, TLR4, IL-1β

## Abstract

**Introduction:**

Rheumatoid arthritis (RA) is an autoimmune disorder characterized by significant disability and teratogenic effects, for which there are few effective curative therapies. Exosomes derived from mesenchymal stem cells (MSCs) exhibit anti-inflammatory and tissue regenerative properties. This study aimed to investigate the therapeutic potential of exosomes derived from human classical interscapular brown adipocytes (hcBAC-exos) in alleviating symptoms of RA in a mouse model.

**Methods:**

We established a mouse model of collagen-induced arthritis (CIA) to evaluate the efficacy of hcBAC-exos. Specifically, we assessed the degree of RA remission by applying vitamin E emulsion, as well as a mixture of vitamin E emulsion and hcBAC-exos, to the foot paws of CIA mice. Additionally, the effects of hcBAC-exos on pro-inflammatory cytokines in macrophages (RAW264.7 cells) were investigated at the cellular level. The active components of hcBAC-exos were analyzed via lipidomics, and the mechanism of their ability to inhibit inflammation was explored.

**Results:**

Administration of hcBAC-exos significantly reduced the expression of pro-inflammatory cytokines in macrophages. In the CIA mouse model, transdermal application of hcBAC-exos led to notable decreases in ankle swelling and the serum levels of IL-1β and TNFα (P < 0.5). Mechanistically, lipidomic analysis showed that Docosahexaenoic acid (C22:6) is highly enriched in hcBAC-exos. Furthermore, we found that C22:6 specifically inhibits IL-1β expression by binding to the amino acids Y183, S210, E265, S182, and R223 of TLR4, mutating these amino acids results in the loss of C22:6 binding activity to TLR4.

**Discussion:**

Our findings suggest that the hcBAC-exos-C22:6-TLR4-IL-1β signaling pathway plays a crucial role in the context of RA, indicating the potential clinical applications of hcBAC-exos in the treatment of inflammatory conditions such as rheumatoid arthritis.

## 1 Introduction

RA is an autoimmune disease characterized by chronic, symmetrical inflammation of the joints (1). The global prevalence of RA ranges from approximately 0.3% to 1%, with the prevalence in women being 3 to 5 times higher than that in men, significantly affecting the quality of life and health status of patients (2). RA is characterized by pathological changes in the joint lining, including infiltration of B cells, macrophages, and CD4^+^ helper T cells into the synovial stroma, which leads to synovial proliferation and subsequent joint swelling and pain (3). Moreover, the overproduction of inflammatory molecules such as tumor necrosis factor (*TNF*), prostaglandin E2 (*PGE2*), interleukin (*IL-1*), and various other cytokines contributes to the persistence of inflammation. In particular, *TNF* and *IL-1* play important roles in the inflammatory process in RA joints (4). Currently, commonly used therapeutic agents for RA include nonsteroidal anti-inflammatory drugs (NSAIDs), steroids, disease-modifying antirheumatic drugs (DMARDs), and biologics (5). While these medications can alleviate the symptoms of RA and slow disease progression, their use is often limited by concerns regarding safety, tolerability, treatment response rates, and cost (6, 7). Consequently, existing treatments for RA fail to adequately address the long-term medication needs of most patients, highlighting the necessity for the development of new therapeutic agents.

Exosomes are vesicles with diameters of 30–150 nm that are secreted by cells under pathophysiological conditions. They play an important role in intercellular communication by influencing the function of recipient cells through the delivery of proteins, lipids, DNA, RNA and other biomolecules (8). In recent years, MSC-derived exosomes have garnered significant attention in the study of RA. Subcutaneous adipose tissue MSC-derived exosomes attenuated symptoms in a mouse model of CIA, and this effect was mediated by IL-1RA in the exosomes (9) On the basis of the ability of exosomes to transmit information to recipient cells, Chen et al. were the first to report that exosomes derived from bone marrow MSCs transfer miR-150-5p to the joint cavity, reversing the migration and invasion of RA fibroblast-like synoviocytes (RA-FLSs) induced by pro-inflammatory factors such as *IL-1β*, *TGF-β*, and *TNFα* by inhibiting the expression of the target genes *MMP14* and *VEGF*, thereby alleviating RA (10). MSCs can be sourced from various tissues, including the umbilical cord, bone marrow and adipose tissue (11). Given that adipose tissue is the largest endocrine organ in the human body, the exploration of adipose tissue-derived exosomes in the context of RA is of significant interest. Mammalian adipose tissue can be categorized into two types, white adipose tissue (WAT) and brown adipose tissue (BAT). BAT is different from WAT in terms of its origin, morphology, function, and secretory factors (12), which leads to varying effects of exosomes from different MSC sources in disease treatment (13–15). Historically, BAT has been recognized for its role in regulating adaptive thermogenesis; however, recent clinical interest has intensified regarding BAT activation as a potential strategy for treating metabolic disorders such as obesity and diabetes (16, 17). Additionally, studies have indicated that BAT transplantation can significantly reduce inflammatory cell infiltration in skin cells affected by localized scleroderma (LoS) (18). Furthermore, a potential association between BAT and RA has been identified, with metformin shown to inhibit CIA progression and improve metabolic dysfunction in obese mice by promoting BAT differentiation (19). Therefore, exosomes derived from BAT may also contribute to the suppression of pro-inflammatory factor expression in RA.

Given the current understanding of RA and the potential role of hcBAC-exos, we focused on their effects on the expression of inflammatory cytokines in macrophages. To identify the active components within hcBAC-exos, metabolomic analysis of the transdermal substances revealed the candidate fatty acid C22:6. *In vivo* studies involved the application of hcBAC-exos and C22:6 to the feet and paws of CIA mice to investigate their effects on RA. By elucidating the mechanisms of action of hcBAC-exos and C22:6 in immune regulation, we aimed to provide significant theoretical and practical insights for the development of novel therapeutic strategies for RA.

## 2 Materials and methods

### 2.1 Cell culture

Human classical interscapular brown adipose tissue was obtained from the interscapular of a spontaneously aborted fetus at Beijing Obstetrics and Gynecology Hospital. The classical interscapular brown adipocytes were isolated as previously described (20, 21). These human classical interscapular brown adipocytes were cultured and expanded in Dulbecco’s modified Eagle’s medium (DMEM) supplemented with 20% fetal bovine serum (FBS), penicillin (100 U/mL), streptomycin (100 μg/mL) and HEPES (20 mM). These cells were used for experiments between passages 5 and 15. RAW264.7 macrophage cell lines and 293FT cell lines were cultured in DMEM supplemented with 10% FBS, penicillin (100 U/mL), and streptomycin (100 μg/mL). All the cells were maintained at 37°C in a 5% CO_2_ environment. The cell lines present in this study were obtained from The American Type Culture Collection.

The study was conducted in accordance with the Declaration of Helsinki, and was approved by the ethics committee of Beijing Obstetrics and Gynecology Hospital, Capital Medical University (protocol code 2023‐KY‐005‐01).

### 2.2 HcBAC-exos isolation and identification

Upon reaching 80% confluency, hcBCA were washed twice with PBS, and the medium was replaced with serum-free medium. After 36 h, the conditioned medium from hcBCA was collected for exosome isolation. The TransExo™ Cell Media Exosome Kit (TransGen Biotech, FE401) was used to isolate hcBAC-exos. Briefly, the cell supernatant was collected and centrifuged at 3000 × g for 30 min at 4°C to remove residual cells and debris. The supernatant was then mixed with EPS-C at the appropriate ratio and allowed to stand at 4°C overnight. The following day, the mixture was centrifuged at 10,000 × g for 30 min at 4°C to precipitate hcBAC-exos. Then PBS was added and the mixture was gently pipetted to collect the hcBAC-exos. The hcBAC-exos were quantified using a BCA protein assay kit and stored at -80°C. The morphology of hcBAC-exos was observed via transmission electron microscopy (USA, FEI). Western blot analysis was conducted to identify the positive proteins (TSG101, CD63 and CD81) of hcBAC-exos. The hcBAC-exos were thoroughly mixed with vitamin E emulsion and applied to the ankle joints of the mice.

### 2.3 Cell uptake analysis *in vitro*

The hcBAC-exos were coincubated with DID dye (Invitrogen, V22887) at 37°C for 30 min. Subsequently, the hcBAC-exos were re-extracted to remove excess dye, resulting in red-labeled exosomes (hcBAC-exos-DID). RAW264.7 cells were inoculated in confocal dishes, and hcBAC-exos-DID was added to the cells for coculturing for 3 and 12 h, respectively. The cells were then fixed with 4% paraformaldehyde for 30 min and washed three times with PBS, after which the nuclei were stained with DAPI working solution. Finally, the uptake of hcBAC-exos-DID by the cells was observed via confocal microscopy.

### 2.4 Plasmid construction and lentiviral packaging

The shRNA oligonucleotide sequences were designed and synthesized via the DSIR website. The synthesized forward and reverse oligonucleotides were annealed to form oligonucleotide duplexes, which were subsequently ligated into the PLKO.1 vector (PLKO.1-shRNA) for lentiviral packaging. Lentivirus can achieve stable transfection. In brief, the core plasmid PLKO.1-shRNA, the packaging plasmid psPAX2, and the envelope plasmid pMD2.G were mixed at a ratio of 4:3:2 in DMEM and co-incubated with three volumes of PEI for 20 min at room temperature. The resulting mixture was added to a cell culture dish containing 70-90% confluent 293FT cells and incubated for 6 h. The mixture was then replaced with fresh basal medium. After 48 h, the cell culture supernatant, which contained the packaged lentiviral particles, was collected. This medium was then added to the target cells to establish a stable cell line.

### 2.5 Mice

Male DBA/1 mice (7–8 weeks old) were obtained from Vital River Laboratory Animal Technology Co., Ltd. All the mice were housed in our SPF laboratory animal facility at the Institute of Zoology, Chinese Academy of Sciences, at a room temperature of 24 °C with a 12 h light/dark cycle. Five mice were housed in each cage with sufficient water and food.

All the animal studies were approved by the Institutional Animal Care and Use Committee of the Institute of Zoology, Chinese Academy of Sciences (protocol code IOZ-IACUC-2023-030).

### 2.6 Induction of collagen-induced arthritis

A collagen-induced arthritis (CIA) mouse model was utilized in this study (22). In brief, complete freund’s adjuvant (CFA) (4 mg/mL) and bovine type II collagen (2 mg/mL) were emulsified at a 1:1 ratio via a homogenizer. On days 0 and 21, 100 μl of emulsified CFA and bovine type II collagen were injected into the tail root of each mouse. Following the second immunization on day 21, hcBAC-exos/C22:6 was administered simultaneously to the ankle joints of the mice. The thickness of the ankle joints in the hind limbs of the mice was measured weekly via a vernier caliper. The clinical arthritis score, assessed on a scale from 0 to3 (0 = normal, 1 = slight swelling and/or erythema, 2 = pronounced swelling, 3 = ankylosis), was calculated as the mean of the scores from the two hind paws for statistics analysis (10, 22). After three weeks, the mice were placed in a chamber containing 3%–4% isoflurane, fully anesthetized within 2–3 minutes, and subsequently euthanized via cardiac puncture bloodletting. The right ankle joints were fixed in 4% neutral buffered formalin for pathological examination.

### 2.7 Quantitative RT-PCR analysis

Total RNA was isolated from cells using TRIzol Reagent (Thermo Fisher Scientific, 15596018CN) and reverse-transcribed with the HiScript III 1st Strand cDNA Synthesis Kit (+gDNA wiper) (Vazyme, R302-01). The expression of mRNA was detected with AceQ Universal SYBR qPCR Master Mix (Vazyme, Q511-02-AA). The list of primers is provided in **Supplementary Table S1** of the Supporting Information.

### 2.8 Western blot

Total protein was extracted via RIPA lysis buffer containing phosphatase and protease inhibitors. The protein concentration was determined using the bicinchoninic acid (BCA) method. Proteins of various molecular weights were subsequently separated via 12% sodium dodecyl sulfate-polyacrylamide gel electrophoresis (SDS-PAGE) and transferred to PVDF membranes. The membranes containing the proteins were incubated overnight at 4°C with the following antibodies: anti-phospho-SAPK/JNK (CST, 4668S), anti-SAPK/JNK (CST, 9252T), anti-IL-6 (Abcam, ab259341), anti-TNFα (Abcam, ab215188), anti-pro-IL-1β (R&D, AF-401-SP), anti-cleaved-IL-1β (CST, 63124), anti-COX2(Abmart, T58852), anti-HSP90(CST, 4874S), anti-CD63(Abmart, M051014), anti-CD81(Abmart, T55724), and anti-TSG101(Abmart, T55985). Next, the PVDF membranes were incubated for 1 h at room temperature with primary and HRP-conjugated secondary antibodies, and the expression levels of each protein were detected using the Enhanced ECL Chemiluminescence Detection Kit (Vazyme, China).

### 2.9 Histology analysis

The right ankle joints of the mice were fixed in 4% paraformaldehyde (PFA) overnight, washed three times with phosphate-buffered saline (PBS) and distilled water for 20 min each, and subsequently transferred to an EDTA decalcification solution for a period of 10–30 days. After dehydration, the ankle joints were embedded in paraffin, sectioned into 4-5 μm slices, and stained with Hematoxylin-eosin staining (H&E) and Safranin O-Fast Green staining (SO/FG). The extent of synovitis and pannus formation was assessed using a graded scale as follows: Grade 0 indicates no symptoms of inflammation; Grade 1 indicates mild inflammation characterized by hyperplasia of the synovial lining; Grade 2 indicates moderate infiltration with noticeable synovial hyperplasia; Grade 3 indicates marked infiltration accompanied by significant synovial hyperplasia; and Grade 4 indicates severe inflammatory cell infiltration and synovial hyperplasia (23).

### 2.10 Protein production and purification

The sequence encoding the extracellular domain of TLR4 (TLR4-ECD) was cloned and inserted into the pMAL-c5X vector, and the resulting recombinant plasmid was transformed into BL21 (DE3) competent cells. The bacteria were cultured at 37°C until the optical density at 600 nm (OD600) reached 0.6-0.8. Isopropyl β-D-1-thiogalactopyranoside (IPTG) was added to a final concentration of 0.8 mM to induce protein expression at 28°C. The bacteria were harvested by centrifugation, resuspended in PBS, and lysed by ultrasonic disruption, and the TLR4-ECD protein was purified using HisTrap FF affinity chromatography.

### 2.11 Molecular docking of TLR4 with C22:6

The atomic coordinates of the extracellular domain of TLR4 (PDB: 2Z64) were obtained from the Protein Data Bank, while the atomic coordinates for C22:6 were sourced from PubChem (CID 445580). The AutoDock Vina program was employed to generate the TLR4-ECD and C22:6, selecting the complex with the lowest energy.

### 2.12 Determination of the Kd value between TLR4 and C22:6

To assess the interaction between TLR4 and C22:6, the dissociation constant (Kd) values were measured using the MST-Nanotemper instrument (Nanotemper, Germany). The principle of the MST-Nanotemper instrument involves generating a microscopic temperature gradient field by heating the sample with an infrared laser, allowing for the assessment of C22:6 -protein binding behavior through the detection of covalently bound fluorescent dyes and the quantification of molecular motions. First, 100 μL of 10 μM TLR4-ECD protein was labeled with a fluorescent dye, followed by sequential dilution of C22:6 in 16 concentration gradients, including 50000 nM, 25000 nM, 12500 nM, 6250 nM, 3125 nM, 1562.5 nM, 781.25 nM, 390.625 nM, 195.3125 nM, 97.65625 nM, 48.828125 nM, 24.4140625 nM, 12.20703125 nM, 6.103515625 nM, 3.0517578125 nM, and 1.52587890625 nM. The protein and C22:6 were then thoroughly mixed at a 1:1 ratio for on-board detection.

### 2.13 Enzyme-linked immunosorbent assay

The levels of IL-1β and TNFα in mouse serum and cellular supernatants were quantified using mouse ELISA kits (CUSABIO, Wuhan) in accordance with the manufacturer’s instructions.

### 2.14 HPLC-MS/MS quantitative Lipidomics

Short-chain fatty acids (SCFAs) were extracted from hcBCA-exos using acetonitrile and subsequently derivatized with 3-nitrophenylhydrazine. SCFAs were analyzed using a Jasper HPLC-Sciex 4500 MD system. Free fatty acids (FFAs) were extracted from exosomes via modified Bligh and Dyer methods. Medium- and long-chain fatty acids were analyzed using a Shimadzu Nexera 20AD-HPLC/ExionLC-AD triple quadrupole/ion trap mass spectrometer (6500 Plus QTRAP; SCIEX). Very-long-chain fatty acids were analyzed via the Jasper HPLC—Sciex 4500 MD system, and FFAs were quantified using d31-FFA 16:0 (Sigma-Aldrich) and d8-FFA 20:4 (Cayman Chemicals) as internal standards (24).

### 2.15 Immunofluorescence

The paraffin-embedded sections were sequentially immersed in xylene and ethanol for dewaxing and hydration. The sections were then treated with antigen retrieval solution, after which endogenous peroxidase activity was blocked with H_2_O_2_. The sections were subsequently incubated in sealing solution for 1 h. After overnight incubation with fluorescent antibodies (CD11b: Invitrogen, 2396712; iNOS: Invitrogen, 53-5920; CD206: Invitrogen, 12-2061), the sections were washed, stained with DAPI, and imaged using a Zeiss LSM 880 confocal microscope. The mean immunofluorescent intensity of each protein was calculated using the software ImageJ (25).

### 2.16 Transdermal drug delivery study

We labeled the carboxyl group of C22:6 with CY5 dye. C22:6-CY5 was mixed with a vitamin E emulsion at a concentration of 0.25 mg/mL and applied to the skin surface of the mice in a dark environment. The control group received a coating of vitamin E emulsion to eliminate autofluorescence. The fluorescence on both the outer and inner surfaces of the skin was measured using a small animal live imager. Skin tissue samples were embedded in optimal cutting temperature (OCT) compound (Tissue-Tek^®^, Sakura Finetek, USA) at -80°C. Sections of 20 μm thick skin were cut using a cryosectioner (CM3050S, Leica, Germany), and images were captured with a Zeiss LSM 880 confocal microscope.

### 2.17 Statistical analysis

The data are expressed as the means ± standard errors (SEs) of the means. Comparisons between groups were performed via one-way ANOVA or Student’s t-test. Statistical significance was set at ***P < 0.001, **P < 0.01 and *P < 0.05.

## 3 Results

### 3.1 Isolation and identification of hcBAC-exos

**Figure 1:**
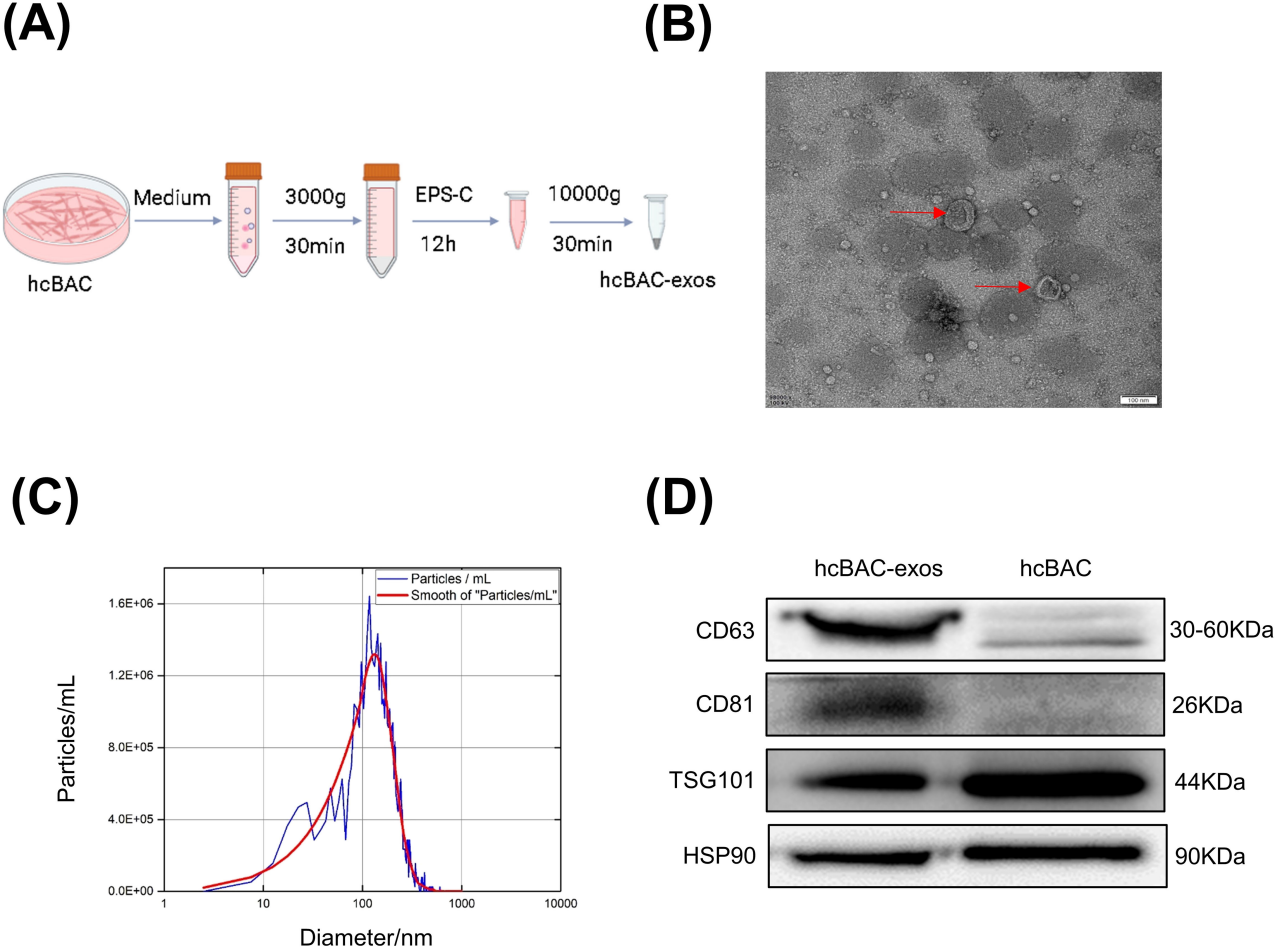
Isolation and anti-inflammatory properties of hcBAC-exos *in vitro*. **(A)** Extraction of hcBAC-exos from brown adipocytes supernatant. **(B)** TEM image showing the morphology of hcBAC-exos. **(C)** Size distribution of hcBAC-exos via NTA. **(D)** Western blot analysis of protein markers associated with hcBAC-exos.

To collect hcBAC-exos, brown adipocytes were cultured in 15 cm diameter dishes. Once the cell confluence reached approximately 80%, the growth medium was replaced with serum-free medium. After 36 h of incubation, the supernatant was harvested, and hcBAC-exos were extracted using an exosome isolation kit according to the manufacturer’s instructions (**Figure 1A**). The morphology and size of the extracted hcBAC-exos were validated through transmission electron microscopy (TEM) and laser scattering microscopy. TEM images demonstrated that hcBAC-exos displayed a characteristic cup-shaped morphology (**Figure 1B**). Nanoparticle tracking analysis (NTA) further revealed that the particle sizes of hcBAC-exos ranged from 50 to 150 nm (**Figure 1C**). Western blot analysis confirmed the specific expression of the exosomal markers CD63 and CD81 (**Figure 1D**). These findings collectively validated the successful isolation of hcBAC-exos.

### 3.2 Anti-inflammatory properties of hcBAC-exos *in vitro*

**Figure 2:**
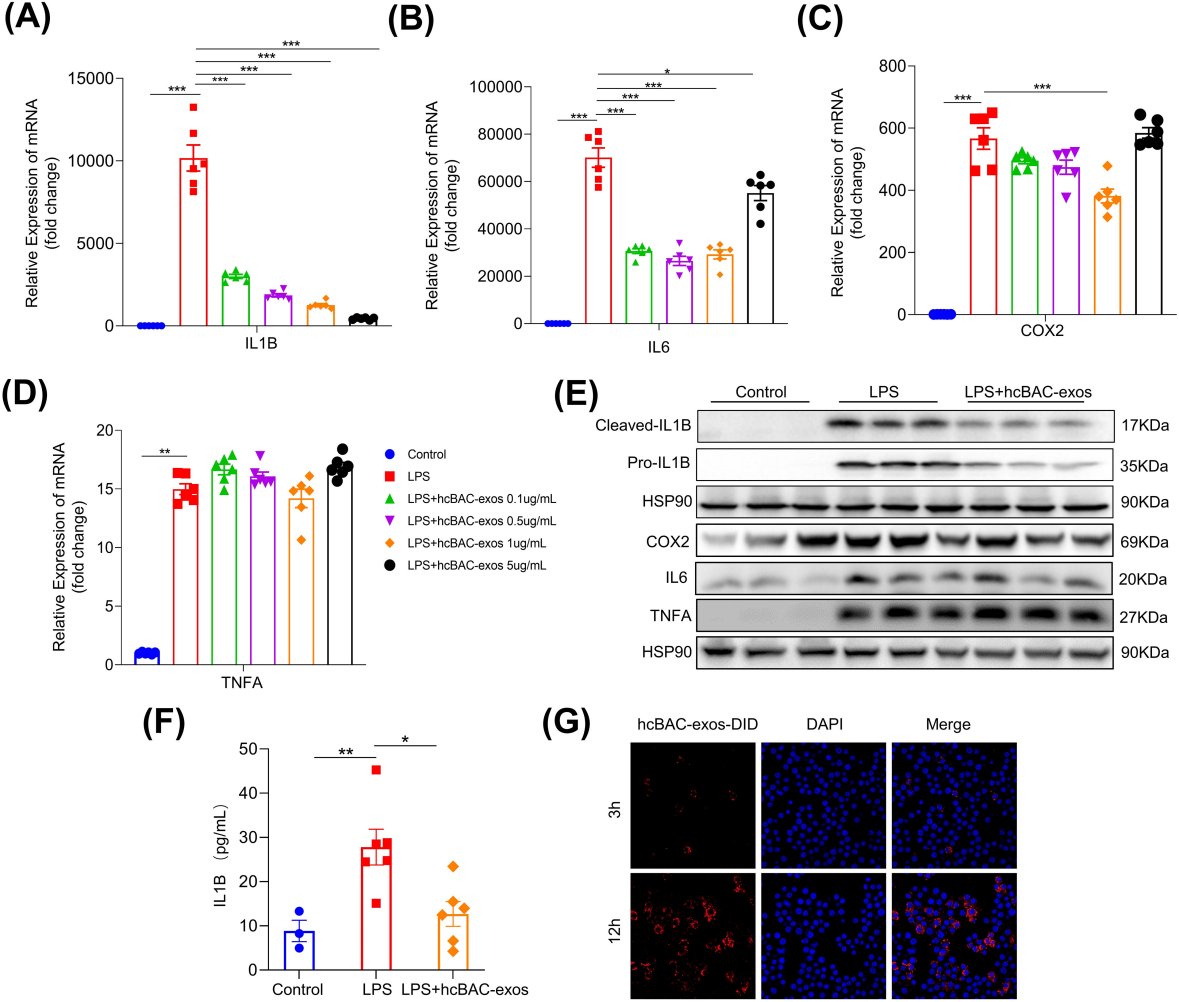
Anti-inflammatory properties of hcBAC-exos *in vitro*. **(A-D)** Effects of hcBAC-exos on the mRNA expression levels of *IL-1β*, *IL-6* and *COX2* in macrophages. **(E)** Effects of hcBAC-exos on the protein expression of pro-inflammatory factors in macrophages. **(F)** Effects of hcBAC-exos on IL-1β in the supernatant of macrophages. **(G)** Confocal microscopy observation of hcBAC-exos-DID uptake by macrophages. Data are shown as mean ± SEM. One-way ANOVA were performed; ***p <0.001, **p < 0.01, and *p < 0.05 was considered to be significant.

Given the pivotal role of macrophages in RA, the RAW264.7 macrophage line was utilized to assess the impact of hcBAC-exos on inflammatory cytokine release. Lipopolysaccharide (LPS) significantly increased the mRNA levels of pro-inflammatory cytokines, including *IL-1β*, *IL-6*, *COX2*, and *TNFα* in macrophages (all P<0.01). Treatment with 1μg/mL of hcBAC-exos notably reduced the mRNA levels of *IL-1β*, *IL-6* and *COX2*, while having no effect on the expression of *TNFα* (**Figure 2A–D**). Consequently, a concentration of 1μg/mL for hcBAC-exos was selected for subsequent cell experiment. Consistently, western blot analysis revealed a significant decrease in IL1-β protein level following hcBAC-exos treatment; however, it had minimal impact on the protein expression levels of IL-6, COX2 and TNFα. (**Figure 2E**). To further evaluate the effect of hcBAC-exos on pro-inflammatory cytokine secretion, an ELISA kit was used to measure IL-1β levels in the supernatant of macrophages, and the results demonstrated that hcBAC-exos markedly inhibited LPS-induced IL-1β secretion (**Figure 2F**). Finally, co-culture experiments using 1,1’-dioctadecyl-3,3,3’,3’-tetramethylindodicarbocyanine,4-chlorobenzenesulfonate salt (DID) dye-labeled hcBAC-exos confirmed the uptake of hcBAC-exos by macrophages, with the uptake levels increasing over time (**Figure 2G**). These results indicate that hcBAC-exos significantly inhibited the expression of IL-1βat the cellular level.

### 3.3 hcBAC-exos improve RA symptoms in CIA mice

**Figure 3:**
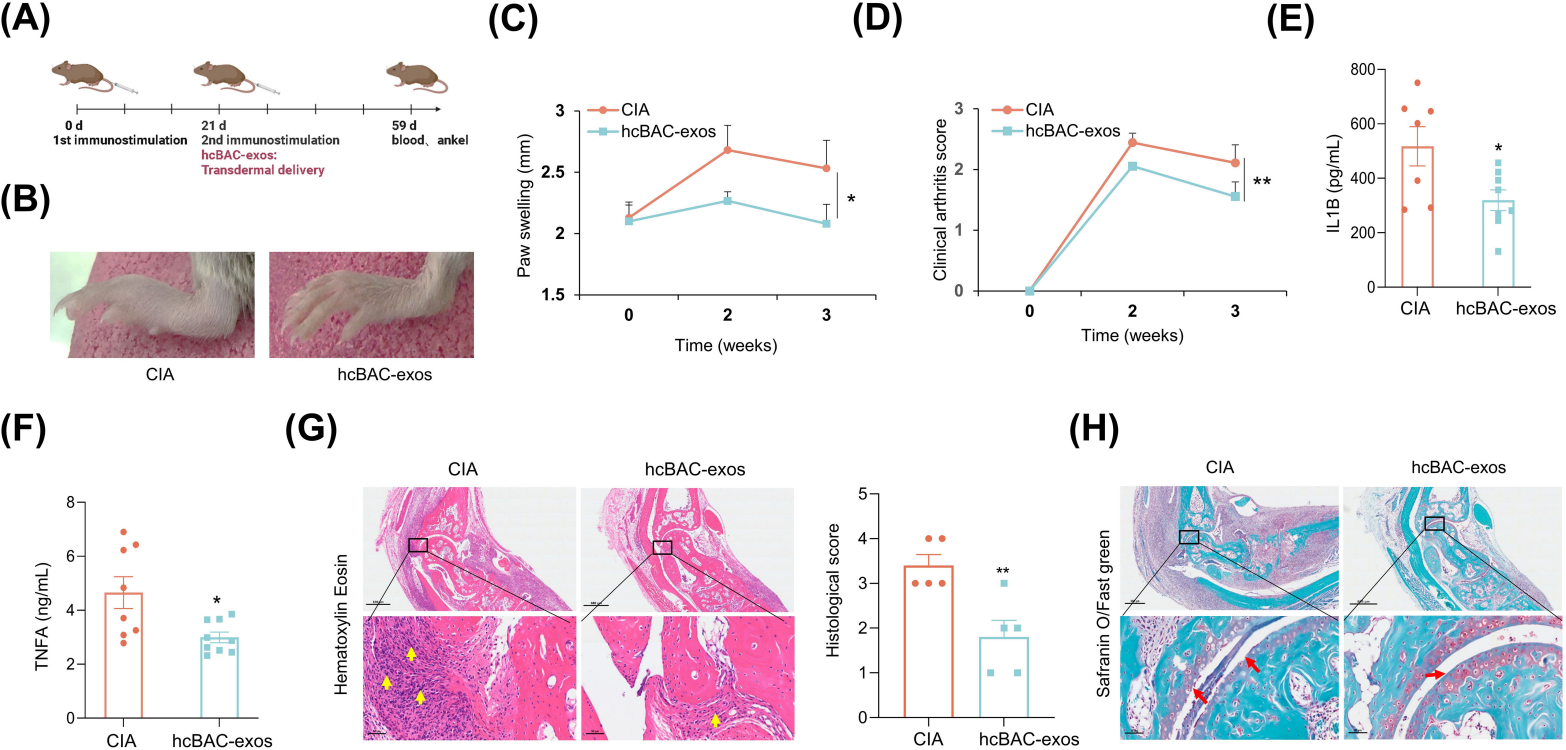
HcBAC-exos alleviate RA symptoms in CIA mice. **(A)** CIA mouse model was generated and administered via protocol. **(B)** Representative images of paw swelling in different experimental groups after hcBAC-exos were applied to the paws of mice for three weeks (n=7-9). **(C)** Measurement of hind paw thickness across various groups of mice (n=7-9). **(D)** Clinical arthritis scores of mice in both groups. **(E, F)** Serum levels of IL-1β and TNFα in the two groups of mice (serum was collected after two weeks of hcBAC-exos application, n=7-9). **(G, H)** Representative histological images of mouse ankles following the application of hcBAC-exos to the paws for three weeks (**G**: H&E staining; **H**: SO/FG staining). Yellow arrows indicate areas of synovial inflammatory cell infiltration and red arrows indicate cartilage locations. Data are shown as mean ± SEM. Two-tailed Student’s t-test and One-way ANOVA were performed; **p < 0.01, *p < 0.05 were considered to be significant.

To assess the transdermal effects of hcBAC-exos on RA *in vivo*, we established a CIA mouse model. The CIA model was established through immunization with collagen type II (CII) and complete freund’s adjuvant (CFA) on day 0, followed by a booster with CII and incomplete freund’s adjuvant (IFA) on day 21. HcBAC-exos, combined with a vitamin E emulsion, were topically administered to the ankle joints and paws of CIA mice, whereas the control group received only the vitamin E emulsion (**Figure 3A**). Three weeks after hcBAC-exos treatment, a significant reduction in swelling of the ankle joints and paws was observed compared with that in the control group (**Figure 3B**). Measurements from the vernier caliper and clinical arthritis scores revealed that the hcBAC-exos group exhibited a notable decrease in paw thickness and arthritis scores when compared to the control group (**Figures 3C, D**, **Supernatant Table 1**). Furthermore, the serum levels of inflammatory cytokines, including IL-1β and TNFα were significantly reduced after hcBAC-exos treatment (**Figures 3E, F**). H&E revealed that inflammatory cell infiltration was dramatically inhibited by hcBAC-exos treatment. Furthermore, SO/FG, which specifically stains chondrocytes, indicated that hcBAC-exos protect against joint chondrocyte damage (**Figures 3G, H**).

### 3.4 FFA in hcBAC-exos suppresses IL-1β expression in macrophages

**Figure 4:**
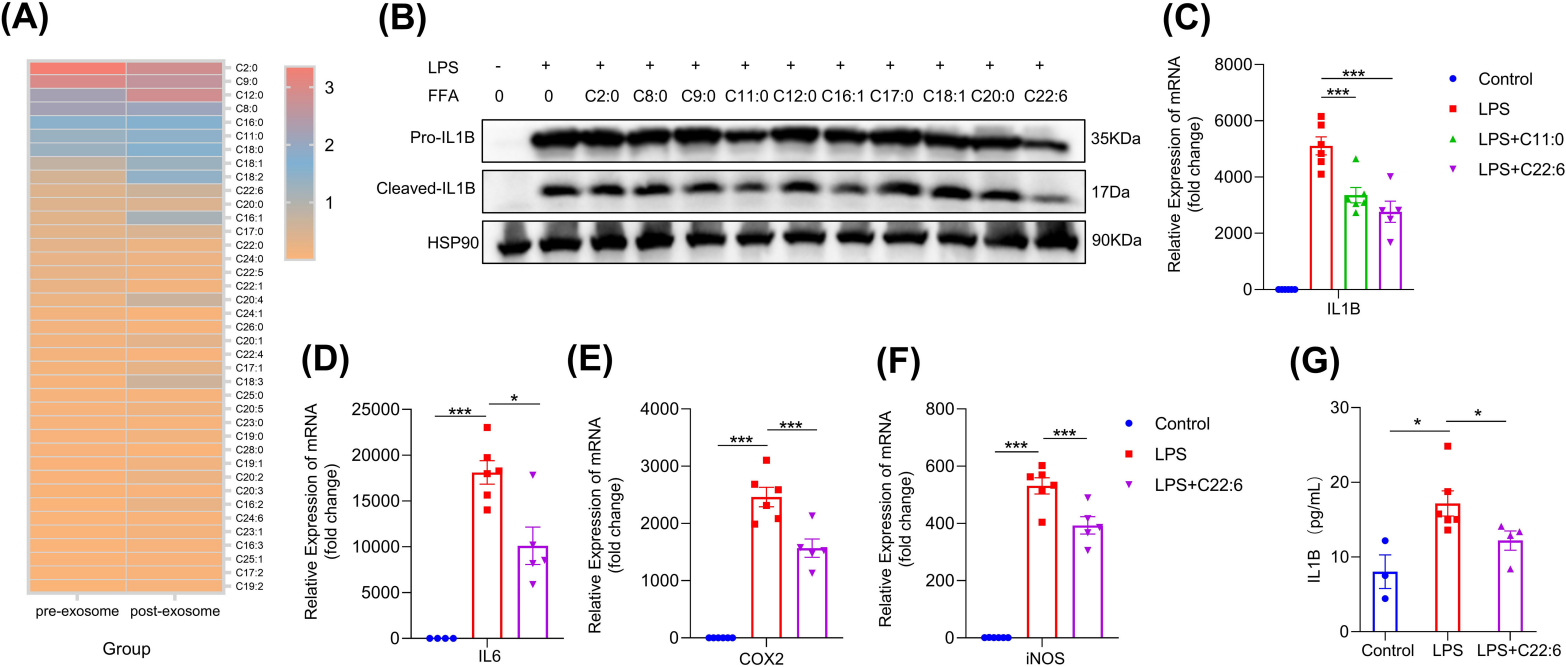
FFA in hcBAC-exos suppresses IL-1β expression in macrophages. **(A)** Heatmap illustrating the types and relative abundances of FFAs in hcBAC-exos before and after transdermal transit (pre-hcBAC-exos: hcBAC-exos before transdermal transit; post-hcBAC-exos: after transdermal transit). **(B)** Effects of FFAs on the protein expression of IL-1β in macrophages. **(C)** Effects of C11:0 and C22:6 on the mRNA expression of IL-1β in macrophages. **(D–F)** Effects of C22:6 on the mRNA expression of IL-6, COX2 and iNOS. **(G)** Effects of C22:6 on IL-1β levels in the supernatant of macrophages. Data are shown as mean ± SEM. One-way ANOVA was performed; ***p <0.001 and *p < 0.05 was considered to be significant.

To identify the key component of hcBAC-exos responsible for inhibiting pro-inflammatory factor expression, we applied hcBAC-exos to the skin of mice and extracted the transdermal substances using a vertical franz diffusion system. Considering the transdermal molecular weight cutoff of 500 Daltons, we analyzed the types and concentrations of free fatty acids present in both the hcBAC-exos and the transdermal material (**Figure 4A**). We found that a considerable amount of free fatty acids was enriched in the post-transdermal portions. To determine which fatty acids are predominantly involved in inflammation, we selected the top 10 fatty acids with high expression levels before and after transdermal processing in the RAW264.7 cell line. Among these fatty acids, C11:0 and C22:6 significantly reduced the protein level of IL-1β (**Figure 4B**). Furthermore, C22:6 exhibited a greater inhibitory effect than C11:0 did (**Figure 4C**). Therefore, we chose C22:6 as our candidate for further investigation. Additionally, C22:6 significantly decreased the mRNA levels of *IL-6*, *COX2*, and *iNOS* (**Figures 4D–F**). Similarly, ELISA analysis of cell supernatants confirmed that C22:6 significantly diminished LPS-induced IL-1β secretion in macrophages (**Figure 4G**). These results highlight C22:6 as a main component of hcBAC-exos that plays a pivotal role in inhibiting the expression of IL-1β.

### 3.5 C22:6 alleviates RA symptoms in CIA mice

**Figure 5:**
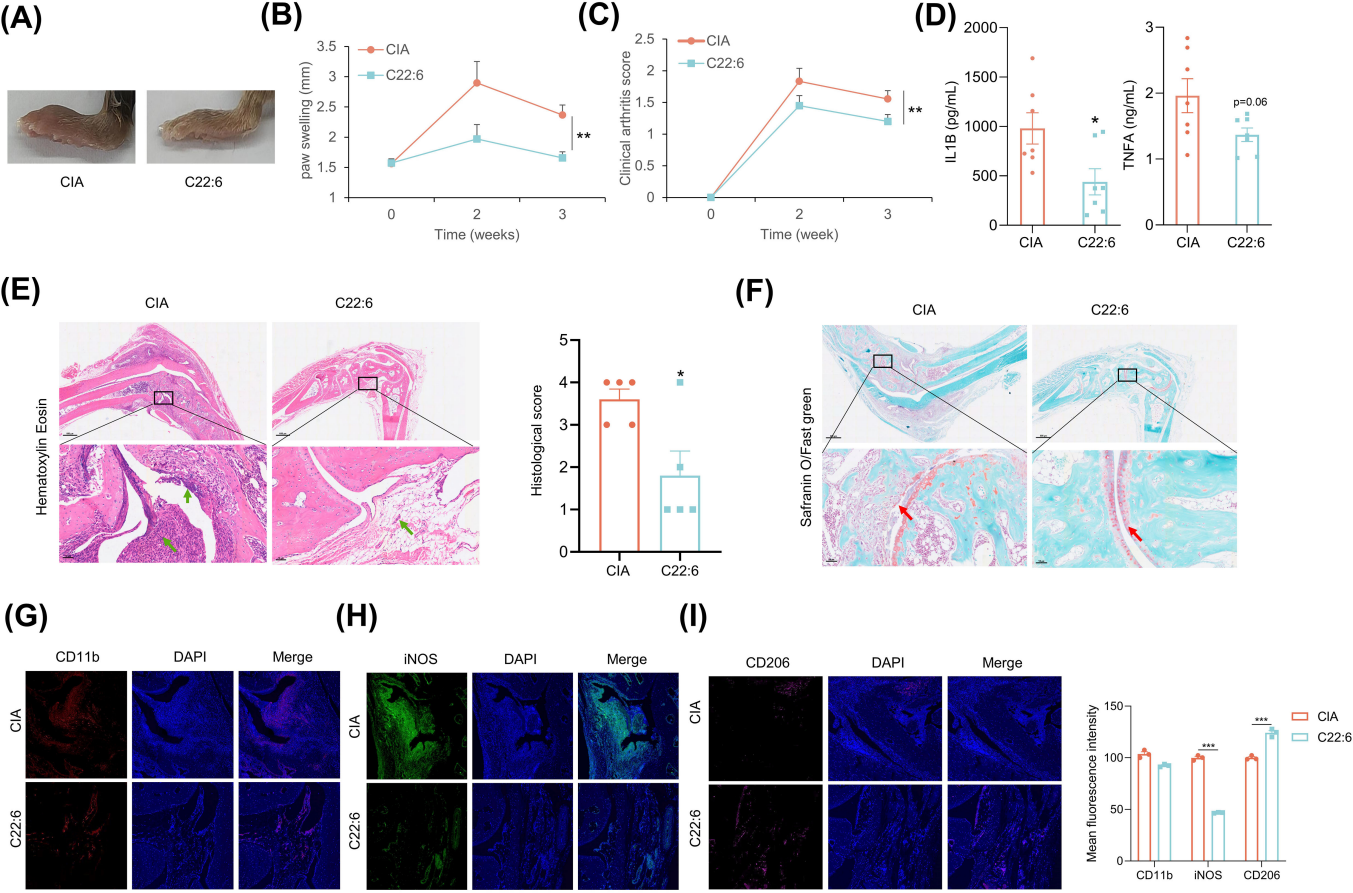
C22:6 improved RA symptoms in CIA mice. **(A)** Representative images of paw swelling in different experimental groups following the application of C22:6 to the paws of mice for three weeks (n=7). **(B, C)** The thickness of the paw and the arthritis score were measured in both the control group and the C22:6 group (n=7). **(D)** Serum levels of IL-1β and TNFa in the two groups of mice, with serum collected after two weeks of C22:6 treatment (n=7). **(E, F)** Representative histological images of mouse ankles after three weeks of C22:6 application (**E**: H&E staining; **F**: SO/FG staining). The green arrows indicate synovial inflammatory cell infiltration, while the red arrows indicate cartilage locations. **(G–I)** Immunofluorescence staining of paraffin sections from mouse ankle joints. (CD11b: total macrophage marker; iNOS: M1 macrophage marker; CD206: M2 macrophage marker). Data are shown as mean ± SEM. Two-tailed Student’s t-test and One-way ANOVA were performed; ***p<0.001, **p < 0.01, *p < 0.05 were considered to be significant.

We further expanded upon our previous findings in a CIA mouse model. C22:6 was administered daily to the ankles and paws of the mice beginning on the second day after immunization. The effect of C22:6 was monitored through weekly assessments of joint thickness. As anticipated, we found that C22:6 significantly reduced paw swelling and arthritis scores after three weeks of treatment (**Figures 5A–C**, **Supernatant Table 2**). In addition, the systemic serum levels of pro-inflammatory factors such as IL-1β and TNFα were markedly lower than those in the control group (**Figure 5D**). Consistently, H&E staining revealed that inflammatory cell infiltration was dramatically inhibited by C22:6 treatment. Furthermore, SO/FG staining demonstrated that C22:6 protects against joint chondrocyte damage (**Figures 5E, F**). Since pro-inflammatory factors are predominantly released by M1 macrophages, we performed immunofluorescence staining of ankle joints. The results revealed a reduction in CD11b+ and significant reduction in iNOS+ (M1 marker) cell populations, and an increase in CD206+ (M2 marker) cells following C22:6 treatment (**Figures 5G–I**). Collectively, these findings suggest that the topical application of C22:6 effectively mitigates the symptoms of CIA in mice.

### 3.6 C22:6 inhibits IL-1β expression via TLR4.

**Figure 6:**
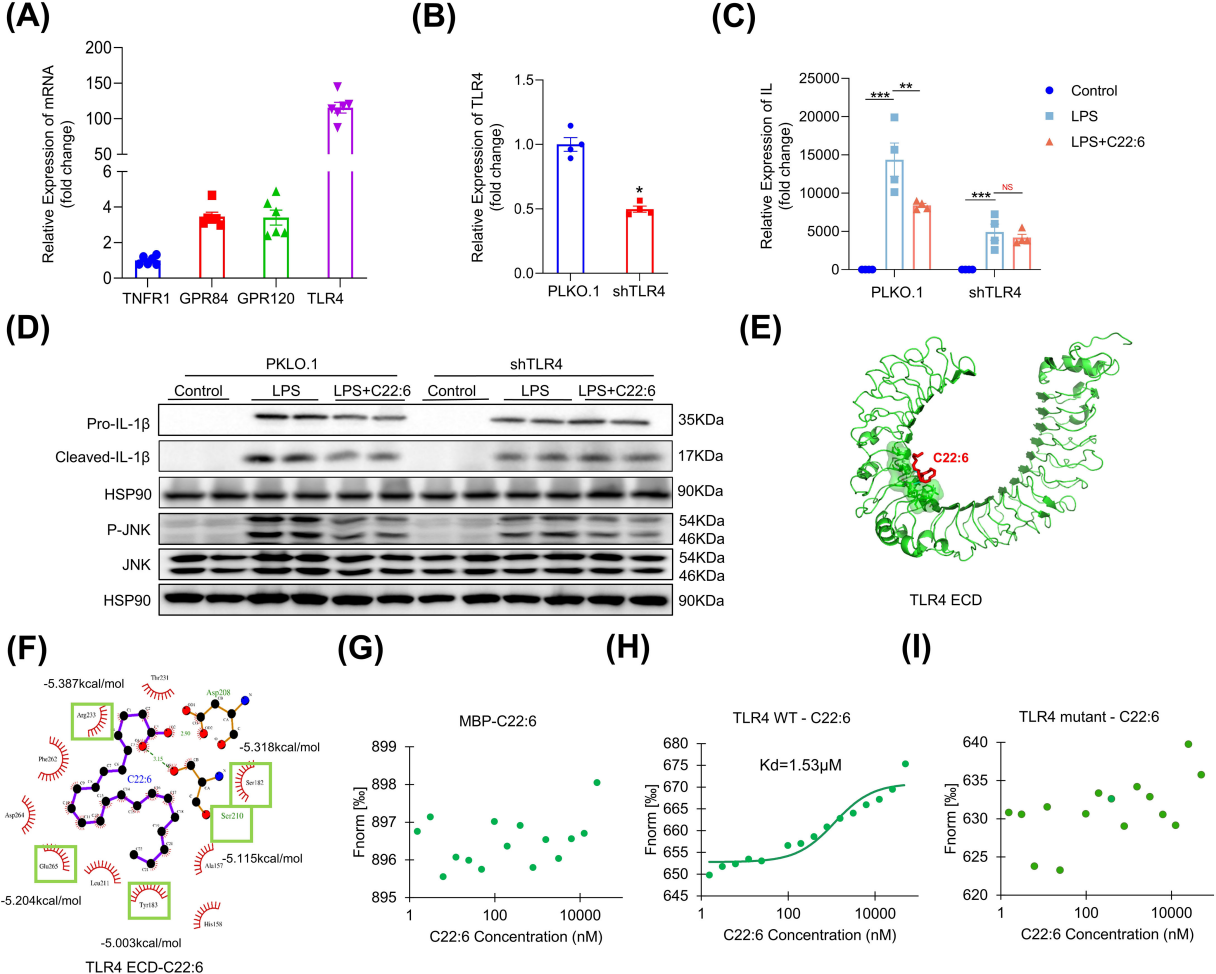
C22:6 inhibits IL-1β expression through TLR4. **(A)** Expression levels of inflammatory and fatty acid-related receptors in RAW 264.7 cells (IL-1R1, GPR40, GPR41, and GPR84 expression levels were too low to be shown). **(B)** shRNA-mediated knockdown of TLR4. **(C, D)** mRNA and protein expression levels of IL-1β in each group were detected by RT-qPCR and WB. **(E, F)** AutoDock Vina software was used to predict potential binding sites for TLR4 and C22:6. **(G-I)** C22:6 affinity assay for MBP, wild-type (WT) TLR4 and the TLR4 mutant. (Fluorescence intensity was measured after mixing varying concentrations of C22:6 with fluorescently labeled MBP **(G)** WT TLR4 **(H)** or the TLR4 mutant **(I)** The x-axis represents the concentration of C22:6, whereas the y-axis represents the fluorescence intensity). Data are shown as mean ± SEM. Two-tailed Student’s t-test or one-way ANOVA were performed; ***p <0.001, **p < 0.01, and *p < 0.05 were considered to be significant. NS represents no significant difference.

The next question is how C22:6 inhibits IL-1β expression. To investigate this, we first assessed the expression levels of fatty acid receptors (*GPR40*, *GPR41*, *GPR43*, *GPR84* and *GPR120*) and inflammatory receptors (*IL1R1*, *TNFR1* and *TLR4*) in macrophages. Notably, *TLR4*, *GPR120*, and *GPR84* exhibited relatively high expression levels compared with *TNFR1* (**Figure 6A**). We then performed loss-of-function experiments, which revealed that the gene expression of *GPR120* and *GPR84* was successfully downregulated after using lentiviral vectors containing shRNAs targeting these receptors. However, knockdown of *GPR120* and *GPR84* did not alter LPS induced IL-1β expression (data not shown). In contrast, after *TLR4* knockdown, C22:6 failed to inhibit LPS induced IL-1β expression (**Figures 6B–D**), indicating that C22:6 may exert its anti-inflammatory effects through TLR4.

To further investigate the interaction between C22:6 and TLR4, we employed AutoDock Vina for molecular docking analysis. Interestingly, we identified 12 potential binding sites for C22:6 on TLR4, with a predicted dissociation constant of -5.585 kcal/mol (**Figures 6E, F**). To confirm the biomolecular interactions, we conducted mutagenesis experiments on the top five residues with high dissociation constants, Y183, S210, E265, S182, and R223 of TLR4, replacing them with alanine. We then purified proteins of both the wild type and mutant forms tagged with myelin basic protein (MBP). We subsequently used the MST-NanoTemper instrument to measure biomolecular interactions. As expected, MBP did not interact with C22:6, whereas wild type TLR4 showed binding affinity with a dissociation constant of 1.53 μM. Interestingly, the mutant TLR4 protein did not bind to C22:6 (**Figures 6G–I**). These findings suggest that C22:6 inhibits inflammation by directly binding to TLR4, specifically by targeting the amino acids Y183, S210, E265, S182, and R223.

### 3.7 Transdermal delivery of C22:6

**Figure 7:**
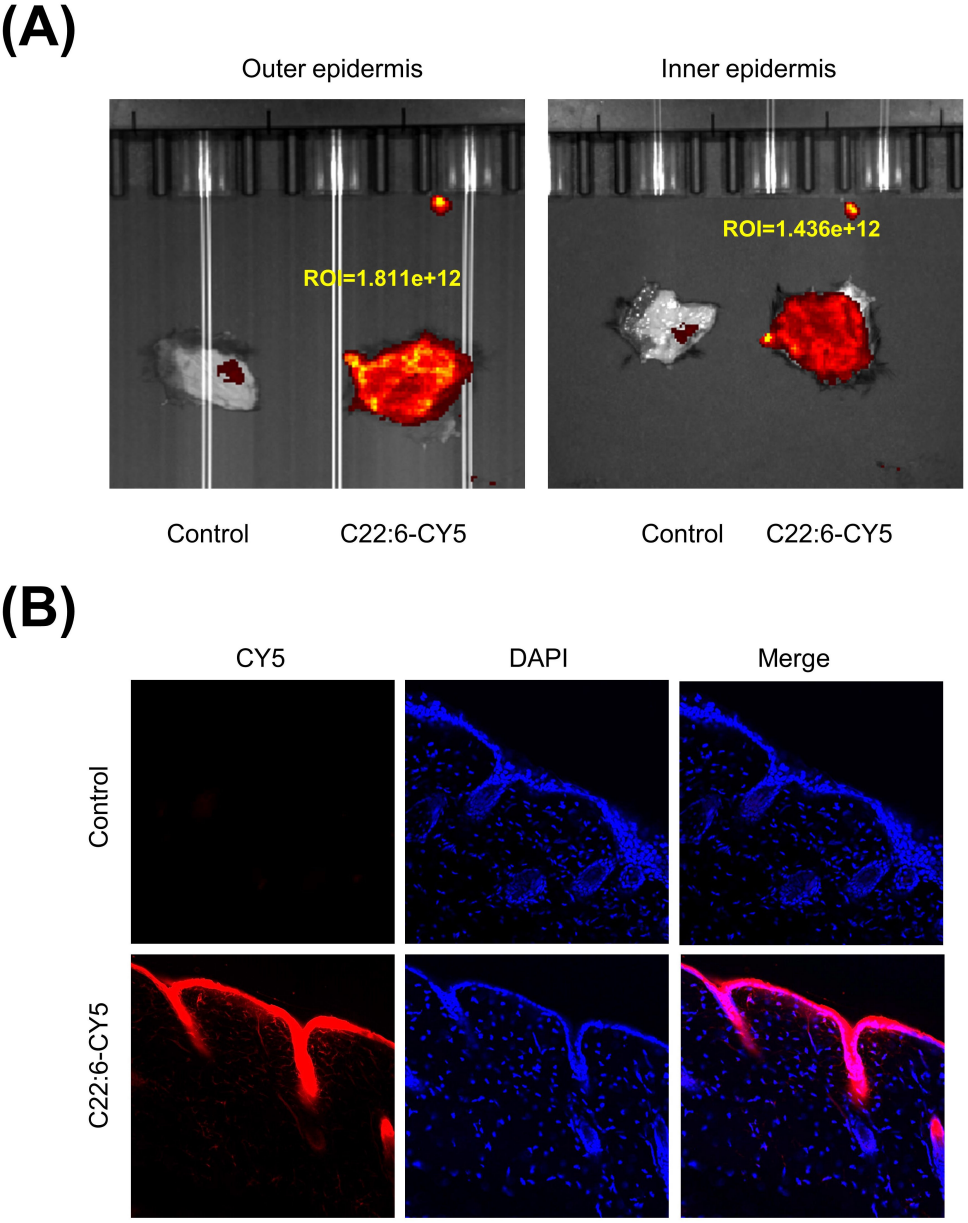
Transdermal delivery of C22:6. **(A)** Fluorescence imaging was used to detect fluorescence values measured on the exterior and interior of the mouse skin. **(B)** Frozen sections of skin tissue were analyzed using fluorescence microscopy to observe the transdermal absorption of C22:6-CY5.

A crucial question is whether C22:6 can penetrate the skin. In recent years, transdermal drug delivery has been increasingly utilized for the treatment of various conditions, including arthritis and skin disorders. This method is noninvasive, allows for flexible dosing, and effectively bypasses first-pass metabolism in the liver (26). To evaluate the transdermal efficacy of C22:6, we conjugated its carboxyl group with a CY5 dye. The C22:6-CY5 complex was mixed with a vitamin E emulsion, applied to the skin of the mice, and shielded from light. Using a small animal live imager, fluorescence was detected both on the skin surface and within the dermis. Both the outer and inner epidermis exhibited high fluorescence intensity (**Figure 7A**). Frozen tissue sections confirmed the presence of red fluorescence in the dermis, indicating successful transdermal absorption of C22:6-CY5 (**Figure 7B**). These results suggest that C22:6 can be effectively delivered transderminally.

## 4 Discussion

In this study, we found that hcBAC-exos suppressed the LPS-induced expression of pro-inflammatory factors in macrophages. Furthermore, the direct application of hcBAC-exos to the skin of the foot paws of CIA mice significantly improved RA symptoms. Lipidomic analysis of hcBAC-exos revealed that the primary component exerting anti-inflammatory effects is C22:6. Mechanistically, we discovered that C22:6 binds directly to the TLR4 receptor, specifically through the amino acids Y183, S210, E265, S182, and R223, thereby suppressing the expression of inflammatory factors. These findings provide new insights into the role of hcBAC-exos in regulating the inflammatory response and suggest potential strategies for RA treatment.

Exosomes are widely present in cell cultures and various body fluids (27, 28). The cell-targeting ability of exosomes enhances their role as drug delivery platforms. Compared with cell-based therapies, exosomes offer advantages such as no cytotoxicity, low immunogenicity, high circulatory stability, and good biocompatibility, making them suitable drug-delivery carriers for various diseases (29). We found that brown adipocytes produce a substantial number of exosomes that can be taken up by macrophages. *In vitro*, hcBAC-exos demonstrated potent anti-inflammatory properties, primarily as evidenced by their ability to inhibit LPS-induced pro-inflammatory cytokine expression in macrophages. This finding aligns with the role of exosomes as cell-to-cell messengers that modulate immune responses and inflammation (30). Moon et al. reported that BAT transplantation significantly reduced bone damage, inflammation, and cartilage damage, along with pro-inflammatory cytokine levels in CIA recipient mice (31). In the present study, we topically applied hcBAC-exos to the skin of the feet and claws of CIA mice, resulting in a significant improvement in RA symptoms. These observations underscore the potential of exosome-based therapies to target specific tissues and modulate local inflammatory responses without systemic side effects. The specificity of exosome action may confer a distinct advantage over traditional broad-spectrum anti-inflammatory drugs, which often have significant side effects (32, 33). Additionally, hcBAC-exos are easier to administer than BAT transplantation and avoid immune rejection, positioning hcBAC-exos as a promising new strategy for RA treatment.

Numerous studies have highlighted the importance of exosomes in intercellular communication by facilitating the transfer of biologically active lipids, proteins, and nucleic acids, thereby influencing the physiological functions of recipient cells (34–36). Wu et al. reported that miR-204-5P was abnormally expressed in the plasma exosomes of patients with RA. Furthermore, the overexpression of miR-204-5p in synovial fibroblasts inhibited their activation by targeting genes associated with cell proliferation and invasion (37). Proteomic analysis of serum exosomes from RA patients revealed that the levels of amyloid A (AA) and lymphatic vessel endothelial hyaluronic acid receptor-1 (LYVE-1) significantly differed between the control and RA groups. These two proteins have potential as additional biomarkers of disease activity in RA patients (38). Furthermore, studies indicate that different types of fatty acids have different mechanisms of action in the regulation of inflammation. Omega-3 fatty acids, such as linolenic acid, eicosapentaenoic acid, and docosahexaenoic acid, can mitigate inflammation by inhibiting the synthesis of inflammatory mediators, including prostaglandins and leukocyte chemokines (39–41). In addition, they modulate inflammation-related signaling pathways, such as the NF-κB and MAPK pathways, thereby exerting an inhibitory effect on inflammation (42). In contrast, omega-6 fatty acids, such as linoleic acid (43), are considered pro-inflammatory. Excessive intake of omega-6 fatty acids enhances the production of inflammatory mediators, such as prostaglandin E2, thereby exacerbating the inflammatory response (44). Consequently, fatty acids derived from BAT-derived exosomes may play a crucial role in the pathogenesis of RA.

We wondered whether some of the fatty acids in hcBAC-exos were involved in inflammatory pathways. To test this hypothesis, we focused on the role of FFAs in hcBAC-exos. Metabolomic analysis revealed that the anti-inflammatory effects of hcBAC-exos were attributed mainly to the presence of C22:6. This finding is important because it highlights the role of specific lipid mediators in the regulation of immune responses. C22:6 directly binds to amino acids Y183, S210, E265, S182, and R223 of the TLR4 receptor, subsequently inhibiting IL-1β expression and providing a clear molecular mechanism by which hcBAC-exos exert their anti-inflammatory effects. Exosomal lipids play crucial roles in the progression of metabolic diseases (45). For example, sphingosine 1-phosphate (S1P), which is found in endothelial-derived exosomes, can enhance the migration of hepatic stellate cells (46). In patients with simple steatosis and early fibrosis associated with nonalcoholic steatohepatitis (NASH), the number of circulating exosomes containing C16:0 ceramides and S1P-rich lipids in plasma gradually increases (47). Currently, the role of lipids in exosomes remains poorly understood, and their role in RA is even less understood. Our understanding of the mechanism by which C22:6 inhibits inflammation through the TLR4 receptor is novel and has broad implications for lipid-mediated immune regulation. Although the application of hcBAC-exos and C22:6 in RA treatment is still in the research stage, their natural biocompatibility and anti-inflammatory properties present promising avenues for non-pharmacological interventions (48).

Stem cell-derived exosomes have been shown to be edited via metabolic glycoengineering (MGE) technology to selectively target macrophages and alleviate symptoms of rheumatoid arthritis (RA) by modulating macrophage heterogeneity (36). The functional resolution and targeted modification of exosomes are important for effective disease treatment. Combining C22:6 with fine-tuned exosomes may facilitate targeted delivery to affected organs, thereby enhancing therapeutic efficacy in suppressing RA. Current research is limited by the use of a single animal model and a single bioactive lipid. Future studies should investigate the role of hcBAC-exos in various RA animal models and explore the synergistic effects of multiple bioactive components within hcBAC-exos. Furthermore, to substantiate our findings, evaluating the potential for clinical translation is essential. The quantification of hcBAC-exos and C22:6, along with their stability during transportation and preservation, as well as their safety and efficacy in human subjects, requires further investigation.

**Figure 8:**
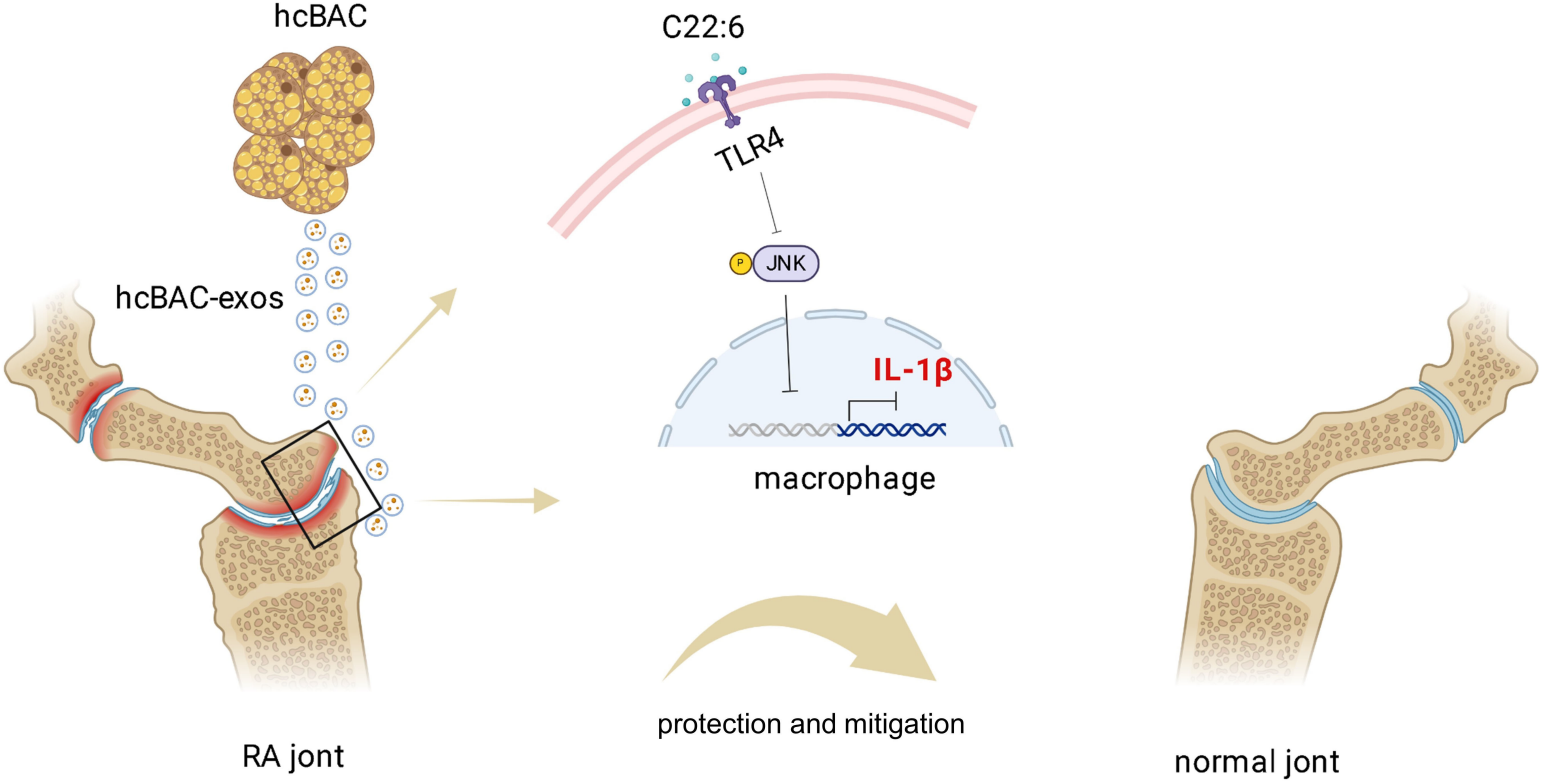
Schematic diagram of the mechanism by which hcBAC-exos operate in RA. The C22:6 component in hcBAC-exos alleviates RA by binding to the TLR4 receptor, thereby inhibiting the expression of IL-1β.

In conclusion, our study revealed the role of hcBAC-exos in modulating the inflammatory response and proposed a potential mechanism of action for hcBAC-exos, especially C22:6, in RA (**Figure 8**). This may represent a novel approach to RA treatment, providing mechanistic insights that establish a foundation for targeted therapy in RA and potentially other inflammatory diseases. Future research should concentrate on addressing the limitations of current studies and translating these findings into clinical practice.

## Data Availability

The original contributions presented in the study are included in the article/**Supplementary Material**. Further inquiries can be directed to the corresponding authors.
