# Supplementary material for: Brown adipocyte exosome - derived C22:6 inhibits the IL-1β signaling pathway to alleviate rheumatoid arthritis
Source: Front Immunol. 2025 May 9;16:1543288. doi: 10.3389/fimmu.2025.1543288 (PMC12098281; doi:10.3389/fimmu.2025.1543288)
Supplement: Supplementary file 1 [file DataSheet1.pdf]

NOTE: Please save this file locally before filling in the table, DO NOT work on the file within your internet browser as changes will not be saved. Adobe Acrobat Reader (available free here) is recommended for completion.

The ARRIVE guidelines 2.0: author checklist

The ARRIVE Essential 10

These items are the basic minimum to include in a manuscript. Without this information, readers and reviewers cannot assess the reliability of the findings.

Item		Recommendation	Section/line number, or reason for not reporting
**Study design**	1	For each experiment, provide brief details of study design including:	
		a. The groups being compared, including control groups. If no control group has been used, the rationale should be stated.	cell：control and treatment group.<br>mice: control (CIA) andtreatment group.
		b. The experimental unit (e.g. a single animal, litter, or cage of animals).	cell: four or six repetitions.<br>mice: cage of mice (each group consisted ten mice)
**Sample size**	2	a. Specify the exact number of experimental units allocated to each group, and the total number in each experiment. Also indicate the total number of animals used.	each group consisted ten mice.<br>total number:forty mice.
		b. Explain how the sample size was decided. Provide details of any *a priori* sample size calculation, if done.	no
**Inclusion and exclusion criteria**	3	a. Describe any criteria used for including and excluding animals (or experimental units) during the experiment, and data points during the analysis. Specify if these criteria were established *a priori*. If no criteria were set, state this explicitly.	cell: remove outliers.<br>mice: individual samples are missing or depleted.
		b. For each experimental group, report any animals, experimental units or data points not included in the analysis and explain why. If there were no exclusions, state so.	cell: remove outliers.<br>mice: individual samples are missing or depleted.
		c. For each analysis, report the exact value of *n* in each experimental group.	cell:four-six.<br>mice:seven-ten.
**Randomisation**	4	a. State whether randomisation was used to allocate experimental units to control and treatment groups. If done, provide the method used to generate the randomisation sequence.	yes<br>The mice were randomly divided into two groups and there was no statistical difference in paw thickness between the two groups.
		b. Describe the strategy used to minimise potential confounders such as the order of treatments and measurements, or animal/cage location. If confounders were not controlled, state this explicitly.	no
**Blinding**	5	Describe who was aware of the group allocation at the different stages of the experiment (during the allocation, the conduct of the experiment, the outcome assessment, and the data analysis).	Rui Jiang:during the allocation;the outcome assessment<br>Yuanyuan Huang:the conduct of the experiment;<br>Wanzhu Jin:, the outcome assessment, and the data analysis;the outcome
**Outcome measures**	6	a. Clearly define all outcome measures assessed (e.g. cell death, molecular markers, or behavioural changes).	cell death、mice death
		b. For hypothesis-testing studies, specify the primary outcome measure, i.e. the outcome measure that was used to determine the sample size.	no
**Statistical methods**	7	a. Provide details of the statistical methods used for each analysis, including software used.	Two groups: T-test; excel<br>Multiple groups: ANOVA; spss
		b. Describe any methods used to assess whether the data met the assumptions of the statistical approach, and what was done if the assumptions were not met.	
**Experimental animals**	8	a. Provide species-appropriate details of the animals used, including species, strain and substrain, sex, age or developmental stage, and, if relevant, weight.	DBA/1;male;7-8 weeks old
		b. Provide further relevant information on the provenance of animals, health/immune status, genetic modification status, genotype, and any previous procedures.	Vital River Laboratory Animal Technology Co Ltd
**Experimental procedures**	9	For each experimental group, including controls, describe the procedures in enough detail to allow others to replicate them, including:	After all mice were purchased, the experimental animal center was housed for one week to acclimatize to the environment. Then the tail root was injected
		a. What was done, how it was done and what was used.	
		b. When and how often.	
		c. Where (including detail of any acclimatisation periods).	
		d. Why (provide rationale for procedures).	
**Results**	10	For each experiment conducted, including independent replications, report:	average and SE.
		a. Summary/descriptive statistics for each experimental group, with a measure of variability where applicable (e.g. mean and SD, or median and range).	
		b. If applicable, the effect size with a confidence interval.	

The Recommended Set

These items complement the Essential 10 and add important context to the study. Reporting the items in both sets represents best practice.

Item		Recommendation	Section/line number, or reason for not reporting
**Abstract**	11	Provide an accurate summary of the research objectives, animal species, strain and sex, key methods, principal findings, and study conclusions.	We established a mouse model of collagen-ind
**Background**	12	a. Include sufficient scientific background to understand the rationale and context for the study, and explain the experimental approach.<br>b. Explain how the animal species and model used address the scientific objectives and, where appropriate, the relevance to human biology.	Rheumatoid arthritis (RA) is an autoimmune disorder characterized by significant disability and teratogenic effects, for which there are few effective curative therapies. This study aimed to investigate the therapeutic potential<br>DBA mice were used to simulate rheumatoid arthritis induced by collagen. DBA mice were the best mouse models for simulating rheumatoid arthritis, with high success rate and high incidence.
**Objectives**	13	Clearly describe the research question, research objectives and, where appropriate, specific hypotheses being tested.	To explore the alleviating effect of exosomes a
**Ethical statement**	14	Provide the name of the ethical review committee or equivalent that has approved the use of animals in this study, and any relevant licence or protocol numbers (if applicable). If ethical approval was not sought or granted, provide a justification.	All the animal studies were approved by the Institutional Animal Care and Use Committee of the Institute of Zoology, Chinese Academy of Sciences. The name of the project is the mechanism of brown adipocytes exosomes in the treatment of rheumatoid arthritis, IOZ-IACUC-2023-209.
**Housing and husbandry**	15	Provide details of housing and husbandry conditions, including any environmental enrichment.	All the mice were housed in our SPF laboratory
**Animal care and monitoring**	16	a. Describe any interventions or steps taken in the experimental protocols to reduce pain, suffering and distress.<br>b. Report any expected or unexpected adverse events.<br>c. Describe the humane endpoints established for the study, the signs that were monitored and the frequency of monitoring. If the study did not have humane endpoints, state this.	no<br>Mouse tail necrosis.<br>no
**Interpretation/ scientific implications**	17	a. Interpret the results, taking into account the study objectives and hypotheses, current theory and other relevant studies in the literature.<br>b. Comment on the study limitations including potential sources of bias, limitations of the animal model, and imprecision associated with the results.	It is plausible that brown fat exosomes alleviate rheumatoid arthritis by inhibiting the expression of inflammatory factors.<br>Current research is limited by the use of a single animal model and a single bioactive lipid. Future studies should investigate the role of hcBAC-exos in various RA animal models and explore the synergistic effects of
**Generalisability/ translation**	18	Comment on whether, and how, the findings of this study are likely to generalise to other species or experimental conditions, including any relevance to human biology (where appropriate).	This study can be extended to human use. First, local administration of the drug on the skin is convenient and does not cause side effects. Secondly, mesenchymal stem cells and their exosomes have been reported to be effective in the treatment of arthritis. Therefore, the clinical promotion of our exosome and its active component C22:6 is
**Protocol registration**	19	Provide a statement indicating whether a protocol (including the research question, key design features, and analysis plan) was prepared before the study, and if and where this protocol was registered.	no
**Data access**	20	Provide a statement describing if and where study data are available.	manuscript and supplementary material
**Declaration of interests**	21	a. Declare any potential conflicts of interest, including financial and non-financial. If none exist, this should be stated.<br>b. List all funding sources (including grant identifier) and the role of the funder(s) in the design, analysis and reporting of the study.	no<br>the National Key Research and Development Program of China (2023YFF1305404), the Open Foundation of The Zhongzhou Laboratory for Integrative Biology (2024KF0001), the General Program (Major
