# Supplementary material for: Brown adipocyte exosome - derived C22:6 inhibits the IL-1β signaling pathway to alleviate rheumatoid arthritis
Source: Front Immunol. 2025 May 9;16:1543288. doi: 10.3389/fimmu.2025.1543288 (PMC12098281; doi:10.3389/fimmu.2025.1543288)
Supplement: Supplementary file 2 [file Table1.docx]

Table S1: List of primers

| Gene Name | Primer Name |
| --- | --- |
| IL-1β | Forward: GGTCAAAGGTTTGGAAGCAG |
|  | Reverse: TGTGAAATGCCACCTTTTGA |
| TNFα | Forward: TGGGCCTCTCATGCACCACC |
|  | Reverse: GAGGCAACCTGACCACTCTCCCT |
| IL6 | Forward: TAGTCCTTCCTACCCCAATTTCC |
|  | Reverse: TTGGTCCTTAGCCACTCCTTC |
| COX2 | Forward: TGCCTGGTCTGATGATGTATG |
|  | Reverse: AGTAGTCGCACACTCTGTTGT |
| iNOS | Forward: CAGGGAGAAAGCGCAAAACAT |
|  | Reverse: CAGGGAGAAAGCGCAAAACAT |
| cyclophilin | Forward: CAAATGCTGGACCAAACACA |
|  | Reverse: GCCATCCAGCCATTCAGTCT |
| GAPDH | Forward: TGGCAAATGGAGATTGTTGCC |
|  | Reverse: AAGATGGTGATGGGGCTTCCCG |
