# Supplementary material for: Brown adipocyte exosome - derived C22:6 inhibits the IL-1β signaling pathway to alleviate rheumatoid arthritis
Source: Front Immunol. 2025 May 9;16:1543288. doi: 10.3389/fimmu.2025.1543288 (PMC12098281; doi:10.3389/fimmu.2025.1543288)
Supplement: Supplementary file 3 [file Table2.docx]

Supernatant Table 1

| group | mouse | Severity score | | |
| --- | --- | --- | --- | --- |
|  |  | Week (0) | Week (2) | Week (3) |
| control | 5319 | 0 | 3 | 3 |
|  | 5320 | 0 | 3 | 3 |
|  | 5322 | 0 | 2 | 1.5 |
|  | 5323 | 0 | 2 | 1 |
|  | 5301 | 0 | 2.5 | 1 |
|  | 5302 | 0 | 2 | 1.5 |
|  | 5303 | 0 | 3 | 3 |
|  | 5305 | 0 | 2 | 3 |
|  | 5306 | 0 | 2.5 | 2 |
| HcBAC-exos | 5359 | 0 | 2 | 1 |
|  | 5360 | 0 | 2 | 1 |
|  | 5361 | 0 | 2 | 1 |
|  | 5362 | 0 | 2.5 | 3 |
|  | 5352 | 0 | 2 | 1 |
|  | 5353 | 0 | 2 | 2 |
|  | 5354 | 0 | 2 | 2 |
|  | 5356 | 0 | 2 | 2 |
|  | 5357 | 0 | 2 | 1 |

Supernatant Table 2

| group | mouse | Severity score | | |
| --- | --- | --- | --- | --- |
|  |  | Week (0) | Week (2) | Week (3) |
| control | 4142 | 0 | 2 | 1.5 |
|  | 4143 | 0 | 2 | 1.5 |
|  | 4144 | 0 | 2 | 1.5 |
|  | 4145 | 0 | 3 | 2 |
|  | 4131 | 0 | 2 | 2 |
|  | 4132 | 0 | 2 | 2 |
|  | 4133 | 0 | 1 | 1 |
|  | 4135 | 0 | 1.5 | 1 |
|  | 4100 | 0 | 1 | 1.5 |
| C22:6 | 4195 | 0 | 2 | 2 |
|  | 4196 | 0 | 1.5 | 1.5 |
|  | 4197 | 0 | 2 | 1 |
|  | 4198 | 0 | 1 | 1 |
|  | 4199 | 0 | 1 | 1 |
|  | 4191 | 0 | 1 | 1 |
|  | 4192 | 0 | 2 | 1 |
|  | 4193 | 0 | 2 | 1.5 |
|  | 4194 | 0 | 1 | 1 |
|  | 4134 |  | 1 | 1 |
